# Biological Insights of Fluoroaryl-2,2′-Bichalcophene Compounds on Multi-Drug Resistant *Staphylococcus aureus*

**DOI:** 10.3390/molecules26010139

**Published:** 2020-12-30

**Authors:** Sally Elmogy, Mohamed A. Ismail, Rabeay Y. A. Hassan, Ahmed Noureldeen, Hadeer Darwish, Eman Fayad, Fahmy Elsaid, Ashraf Elsayed

**Affiliations:** 1 Botany Department, Faculty of Science, Mansoura University, Elgomhouria St., Mansoura 35516, Egypt; sally_elmogy@yahoo.com; 2 Chemistry Department, Faculty of Science, Mansoura University, Elgomhouria St., Mansoura 35516, Egypt; mismail@mans.edu.eg; 3 Nanoscience Program, University of Science and Technology (UST), Zewail City of Science and Technology, 6th October City, Giza 12588, Egypt; ryounes@zewailcity.edu.eg; 4 Applied Organic Chemistry Department, National Research Centre (NRC), Dokki, Giza 12622, Egypt; 5 Department of Biology, College of Sciences, Taif University, P.O. Box 11099, Taif 21944, Saudi Arabia; a.noureldeen@tu.edu.sa; 6 Department of Agricultural Zoology, Faculty of Agriculture, Mansoura University, Elgomhouria St., Mansoura 35516, Egypt; 7 Department of Biotechnology, College of Sciences, Taif University, P.O. Box 11099, Taif 21944, Saudi Arabia; hadeer@tu.edu.sa (H.D.); e.esmail@tu.edu.sa (E.F.); 8 Department of Medicinal and Aromatic Plants, Horticulture Institute, Agriculture Research Center, Giza 12619, Egypt; 9 Biology Department, Faculty of Science, King Khalid University, P.O. Box 10347, Abha 61321, Saudi Arabia; felsaid@kku.edu.sa; 10 Zoology Department, Faculty of Sciences, Mansoura University, Elgomhouria St., Mansoura 35516, Egypt

**Keywords:** bichalcophene derivatives, *S. aureus*, antibacterial activity, anti-biofilm agents, biosensors

## Abstract

Resistance of bacteria to multiple antibiotics is a significant health problem; hence, to continually respond to this challenge, different antibacterial agents must be constantly discovered. In this work, fluoroaryl-2,2′-bichalcophene derivatives were chemically synthesized and their biological activities were evaluated against *Staphylococcus aureus* (*S. aureus*). The impact of the investigated bichalcophene derivatives was studied on the ultrastructural level via scanning electron microscopy (SEM), molecular level via sodium dodecyl sulfate-polyacrylamide gel electrophoresis (SDS-PAGE) method and on the biofilm inhibition via the electrochemical biosensors. Arylbichalcophenes’ antibacterial activity against *S. aureus* was affected by the presence and location of fluorine atoms. The fluorobithiophene derivative **MA-1156** displayed the best minimum inhibitory concentration (MIC) value of 16 µM among the tested fluoroarylbichalcophenes. Over a period of seven days, *S. aureus* did not develop any resistance against the tested fluoroarylbichalcophenes at higher concentrations. The impact of fluoroarylbichalcophenes was strong on *S. aureus* protein pattern showing high degrees of polymorphism. SEM micrographs of *S. aureus* cells treated with fluoroarylbichalcophenes displayed smaller cell-sizes, fewer numbers, arranged in a linear form and some of them were damaged when compared to the untreated cells. The bioelectrochemical measurements demonstrated the strong sensitivity of *S. aureus* cells to the tested fluoroarylbichalcophenes and an antibiofilm agent. Eventually, these fluoroarylbichalcophene compounds especially the **MA-1156** could be recommended as effective antibacterial agents.

## 1. Introduction

Several antibiotics have been developed to control bacterial infections after penicillin discovery [1]. However, the resistance of bacteria to several antibiotics has developed and become a major global problem [2]. The resistance development rate is affected by several factors, including excessive use and abuse of antibiotics [3]. To restrict the resistant bacteria spreading, different effective therapies need to be continuously developed in order not to return to the pre-antibiotic therapy. Such problems have led to the need to use chemically synthesized heterocyclic compounds that can be associated with wide-spectrum activity and have a substantially lower propensity than antibiotics to cause microbial resistance [4].

Most biomass cellulose and related products, various pharmaceutical products, all nucleic acids, many natural and synthetic dyes, are heterocyclic compounds. Fifty-nine percent of US FDA-approved medications contain nitrogen heterocycles. [5]. Sulfur, oxygen, and nitrogen are still the most prevalent heteroatoms. Cationic heterocyclic compounds containing two units of thiophene and/or furan are called bichalcophenes. Such compounds have shown a wide variety of biological activities [6,7,8]. One bifuran derivative of a bichalcophene series was more effective against methicillin-resistant *S*. *aureus* than the antibiotic vancomycin in mice [9]. A series of phenyl bichalcophenes were found to have an efficient antimicrobial activity against Gram-negative bacteria, *E. coli* and *P. aeruginosa* in addition to Gram-positive bacteria, *S. aureus* and *B. subtilis* and some strains of fungi such as *Saccharomyces cerevisiae* [10]. Due to the promising antimicrobial activity of these bichalcophene derivatives, the relationship between the novel bichalcophenes and tetracycline was explored in previous reports [11].

For better pharmacological properties, greater antimicrobial activity and lower toxicity than their non-fluorinated parent compounds, a series of fluoroarylbichalcophenes has been synthesized and published. These fluoroarylbichalcophene derivatives have been tested for their toxic effect on *S. typhimurium* TA1535 viability. It was found that, all the investigated fluorine-containing bichalcophenes exhibited a significant reduction on the *S. typhimurium* TA1535 viability at 50 and 100 µM. Moreover, it has been found that these investigated compounds were acted as potent antimutagenic [12] and anticancer agents [13]. The presence of fluorine atoms generally increases lipophilicity and therefore biological availability [14]. Fluorine substitution, through microsomal inhibition, was found to suppress mutagenicity of quinolone [15]. The enzymatic oxidation is usually prevented at the F-substitution site if the aromatic nucleus is fluorinated due to its electron-negativity nature [16]. As a result, F-substitution at the activation site may reduce the aromatic compounds carcinogenicity [17,18] and mutagenicity [19]. At position 3, quinoline was deprived of genotoxicity in vitro [20] and in vivo, while fluoroquinoline was genotoxic as quinolone [21]. The biological and chemical properties of compounds can be altered due to the fluorine substitution of molecule and this can lead to the development of a massive number of novel fluorinated drugs. The high electronegativity of the fluorine substituent affected a molecule metabolism, distribution, and absorption of molecules by modifying the electron distribution in that molecule. Pharmaceutical products containing fluorine are used in a variety of fields for the manufacture of anti-inflammatory, anticancer pharmaceutical drugs, cardiac therapy, anti-parasitic and antibiotics and general anesthetics [14]. Due to the exposure of some encouraging reports concerning the biological activity of fluorophenylbichalcophene derivatives, this research reported herein was launched to investigate the antibacterial impacts of monocationic fluorinated bichalcophenes at the molecular, cellular ultrastructure and biosensing levels against *S. aureus*.

## 2. Results

### 2.1. Antimicrobial Activity of the Tested Aryl-2,2′-Bichalcophene Derivatives

**Figure 1:**
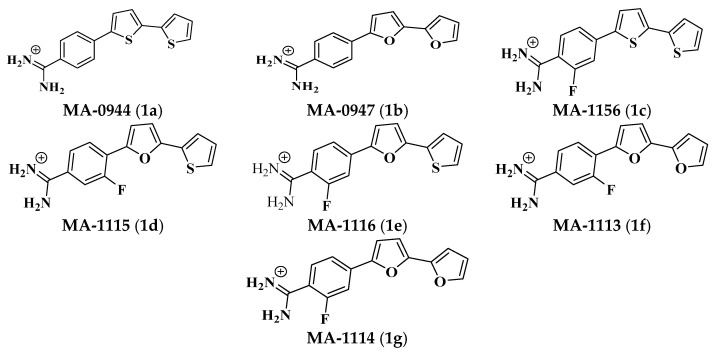
Chemical structures of monocationic bichalcophene derivatives.

**Figure 2:**
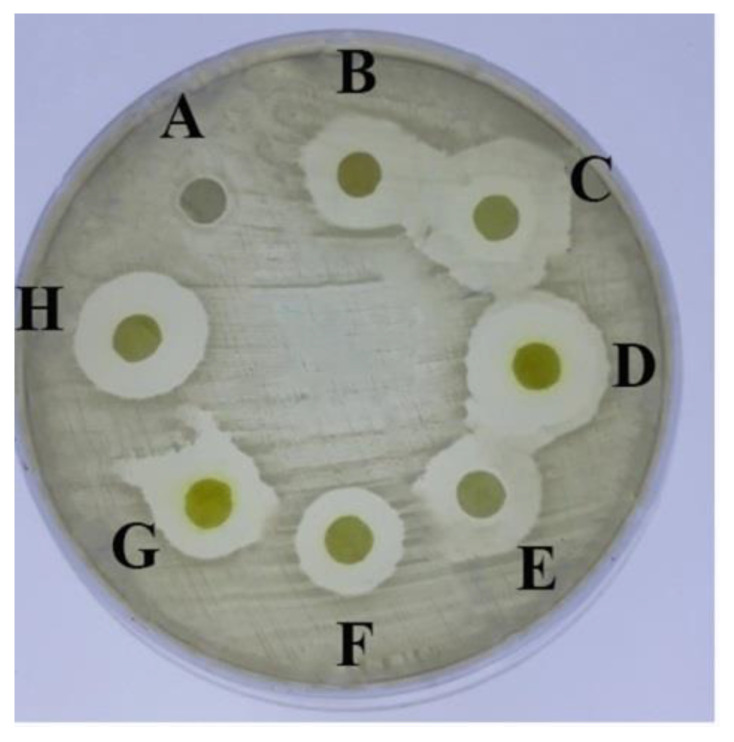
Susceptibility of *S. aureus* towards bichalcophenes, on Mueller–Hinton agar medium incubated at 37 °C for 24 h. (A) DMSO, (B) **MA-1113**, (C) **MA-1114**, (D) **MA-1115**, (E) **MA-1116**, (F) **MA-1156**, (G) **MA-0944**, (H) **MA-0947**. Note: The results expressed as zone inhibition in mm diameter. Assays were performed in triplicate exposing the mean (mean ± SE). Abbreviation: SE, standard error.

The tested bichalcophenes (Figure 1a–g) exhibited a wide range of antimicrobial activity against *S. aureus*, whereas the maximum inhibition zone diameter recorded 20 mm with **MA-1115** and **MA-1114** compounds equally while the zone of inhibition produced by the non-fluorinated parent compounds, **MA-0944** and **MA-0947** were 14 mm and 15.5 mm diameter respectively. Thus, the **MA-1156** compound has a considerable antimicrobial activity with inhibition zone diameter 15 mm. **MA-1116** and **MA-1113** showed an equal antimicrobial activity with inhibition zones diameter 16 mm as shown in Figure 2 and Table 1. The growth inhibition was not observed around the control disc containing dimethyl sulfoxide (DMSO). The antimicrobial activity of the tested compounds with standard antibiotics as Cefoxitin and Gentamycin was compared using the disc diffusion method (data not shown). The antibiotics exhibited antimicrobial activities was very close to the activity of the parent compounds but in case of fluoroarylbichalcophenes the activity was lower.

### 2.2. Minimum Inhibitory Concentrations (MIC) of the Tested Fluoroaryl-2,2′-Bichalcophene Derivatives

The MIC value of the tested fluoroarylbichalcophene derivatives was recorded at a minimum concentration not showing microbial growth (Table 2). Compound **MA-1156** was the most effective candidate showing the best MIC value of 16 µM. The **MA-1115** compound demonstrated a potent antimicrobial activity with the MIC value of 32 µM. **MA-1116** compound prevented the growth of *S. aureus* at 64 µM. On the other hand, the **MA-1113** and **MA-1114** compounds displayed the highest MIC values at 128 µM and subsequently the lowest activity.

### 2.3. Structure-Activity Relationship (SAR)

The observed antibacterial activity of the **MA-1156** indicated that the bithiophene linked to fluorophenyl moiety is promoting the antibacterial activity. Introducing fluorine into the phenyl group of compound **MA-0944** increased its activity from MIC 12–16 μM. However, replacement of the thionyl moiety by a furyl moiety decreased the activity from MIC 16–32 µM.

### 2.4. Detection of the Tested Fluoroaryl-2,2′-Bichalcophenes-Resistant Variants

**Figure 3:**
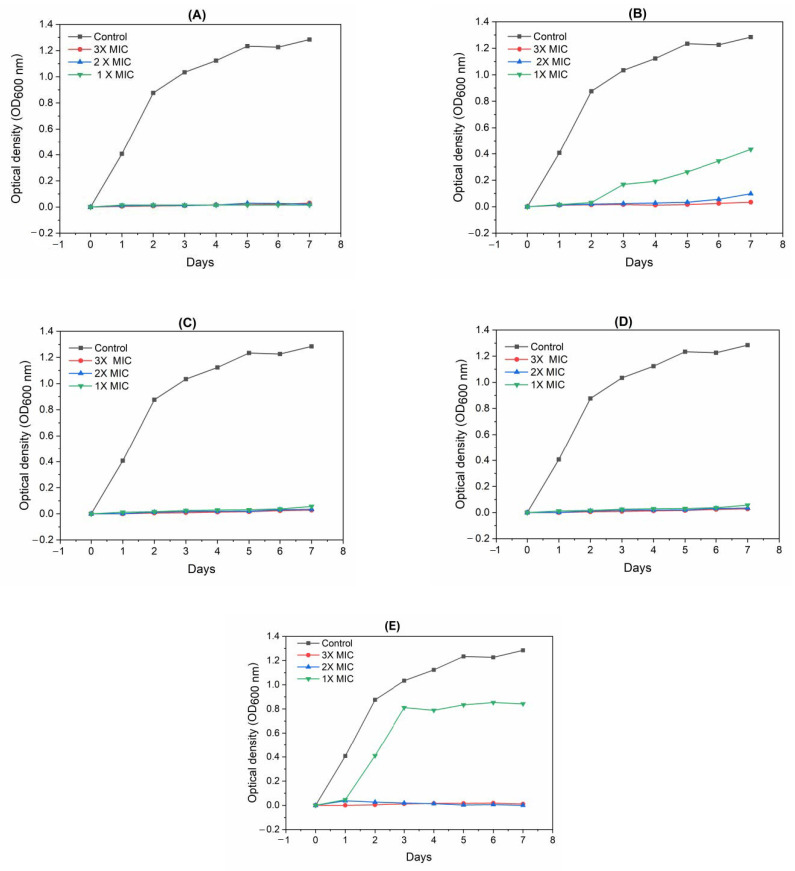
Development of resistance by *S. aureus* in the presence of the tested fluoroarylbichalcophenes at (3, 2, and 1× MIC) during 7 days of incubation. (**A**) **MA-1156** compound; (**B**) **MA-1115** compound; (**C**) **MA-1116**; (**D**) **MA-1113**; (**E**) **MA-1114**.

The compounds **MA-1156**, **MA-1116**, and **MA-1113** could effectively prevent *S. aureus* growth even after seven days with all concentrations. In contrast, one case of resistance was developed against the compound **MA-1115** after the 3rd day of incubation with one-fold concentration (1× MIC). Another case of resistance appeared with one-fold concentration (1× MIC) of the compound **MA-1114** after the 2nd day of incubation whereas the bacteria could not develop any resistance at higher concentrations (2× and 3× MIC) (Figure 3).

### 2.5. Effect of Fluoroarylbichalcophenes on *S. aureus* Protein Pattern

**Figure 4:**
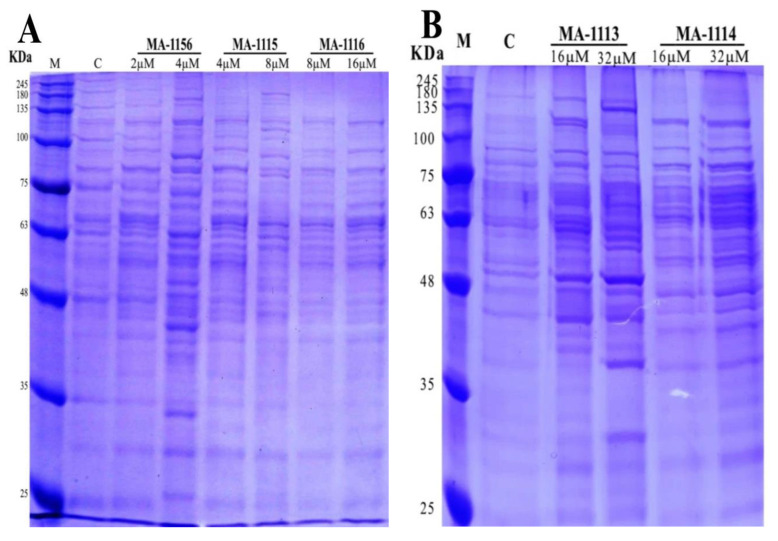
Protein pattern of *S. aureus* cultivated on Luria–Bertani (LB) broth media, (**A**) treated with **MA-1156** (2 and 4 µM), **MA-1115** (4 and 8 µM) and **MA-1116** (16 and 32 µM) M = marker, C = control or untreated bacteria; (**B**) treated with **MA-1113** (16 and 32 µM) and **MA-1114** (16 and 32 µM).

The changes of protein banding of *S. aureus* treated with the two sub-MIC concentrations of the tested fluoroarylbichalcophenes were presented in Figure 4. The total number of bands for **MA-1156**, **MA-1115** and **MA-1116** recorded 36 distributed as: 13 polymorphic, 12 monomorphic and 11 unique bands (Figure 4A). For the control sample, the total number of bands was 22 missing unique bands. Lane of 4 µM **MA-1156** recorded the 27 maximum bands number higher than the control including eight unique bands. On the other hand, the 19 minimum bands number exposed at both lanes of 8 and 16 µM **MA-1116**. The lane of 2 µM **MA-1156** showed 22 total bands similar to the control. Slight changes compared to control appeared in a lane of 4 µM **MA-1115** recording 21 total protein bands and no unique bands were observed. Lane of 8 µM **MA-1115** recorded the same 22 total protein bands as the control.

For **MA-1113** and **MA-1114**, the total number of bands recorded as 30 distributed as: 11 polymorphic, 17 monomorphic, and two unique bands (Figure 4B). For the control, the total number of bands was 22 with no unique bands. Two lanes of 16 and 32 µM **MA-1113** increased the total protein to 26 higher than control. The 21 minimum band numbers exposed at lanes of 16 and 32 µM **MA-1114**.

### 2.6. Changes in Cellular Ultrastructure of S. aureus Treated with One Sub-MIC of Fluoroarylbichalcophenes

**Figure 5:**
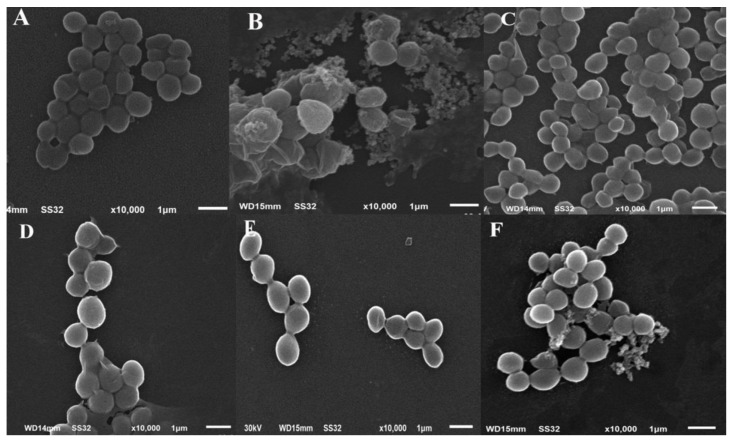
SEM micrographs of control *S. aureus* cells (**A**) *S. aureus* cells treated with **MA-1156** (**B**), **MA-1115** (**C**), **MA-1116** (**D**), **MA-1113** (**E**) and **MA-1114** (**F**).

Based on the results of antimicrobial activities, *S. aureus* was selected for scanning electron microscopy in the presence of the investigated fluoroarylbichalcophenes. The micrographs of the control untreated *S. aureus* cells showed their normal morphology as typical grapelike cluster arrangement. The cells were intact, around 1 µm in diameter and looked smooth rounded in shape (Figure 5A). On the other hand, after incubation with a sub-MIC, some bacteria exhibited different morphological abnormalities. Treatment with 4 µM of **MA-1156** caused random aggregations of sticky cells having smaller size around 0.7 µm diameter with wavy cell walls. SEM micrographs of *S. aureus* cells treated with both 8 µM of **MA-1115** and 16 µM of **MA-1116** showed random clusters appeared to be smaller in diameter (0.5–0.8 µm) than the control sample. For treatment with 16 µM of **MA-1116**, linear arrangement was observed. In case of 32 µM of **MA-1113** treatment, the cells of *S. aureus* exhibited abnormal shape of few numbered and smaller cells (nearly 0.8 µm in diameter) arranged in clusters with less width and length. Treatment with 32 µM of **MA-1114** showed a decrease in cells number varying markedly in size and shape. Many cells were smaller (0.6–0.9 µm diameter) than usual (Figure 5).

### 2.7. Cell Viability Determination Using WST-1 Reagent

The effect of the tested fluoroarylbichalcophenes on the microbial activity was studied by monitoring the growth rate via optical density as a measure of the cell number. However, the rapid detection of these bichalcophenes antibacterial effects on the cell viability rather than the cell number is still needed. Therefore, WST-test was implemented. The reduction of tetrazolium salt WST-1 by the viable *S. aureus* cells results in a water-soluble yellow formazan, which can be easily quantified at the wavelength of 450 nm.

**Figure 6:**
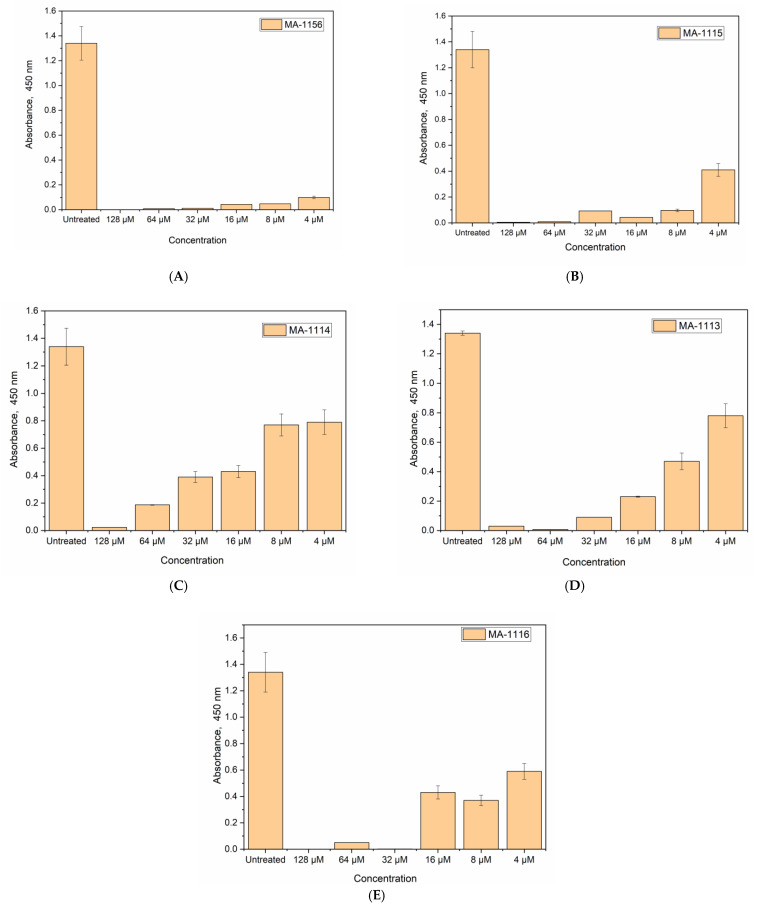
Testing the cell viability of *S. aureus* treated with the tested fluoroarylbichalcophenes at different concentrations (0–128 µM) using the WST1-test (**A**) compound **MA-1156**; (**B**) compound **MA-1115**; (**C**) compound **MA-1116**; (**D**) compound **MA-1113**; (**E**) compound **MA-1114**.

As depicted in Figure 6, *S. aureus* was most sensitive to **MA-1156**, since the cell viability was strongly inhibited over all concentrations (viability%: 7.4%, 3.5%, 3.2%, 0.8%, 0.6%, and 0). On the other hand, the sensitivity of *S. aureus* to **MA-1114** was the lowest among the tested compounds as the treated *S. aureus* cells were viable over all concentrations except 128 µM at which the cells were not viable with 1.7% viability. For **MA-1115**, all concentrations were lethal to *S. aureus* cells except the lowest concentration 4 µM at which the bacterial cells were viable with 30% when the bacterial strain was treated. **MA-1116** and **MA-1113** had the same potent effect on *S. aureus* cells. Some concentrations 128, 64, and 32 µM were lethal, while the cells treated with 16, 8, and 4 µM were viable with percentage 44%, 27.6%, and 32% for **MA-1116** and 58%, 35%, and 17% for **MA-1113** respectively.

### 2.8. Sensing the Response of *S. aureus* Biofilms

**Figure 7:**
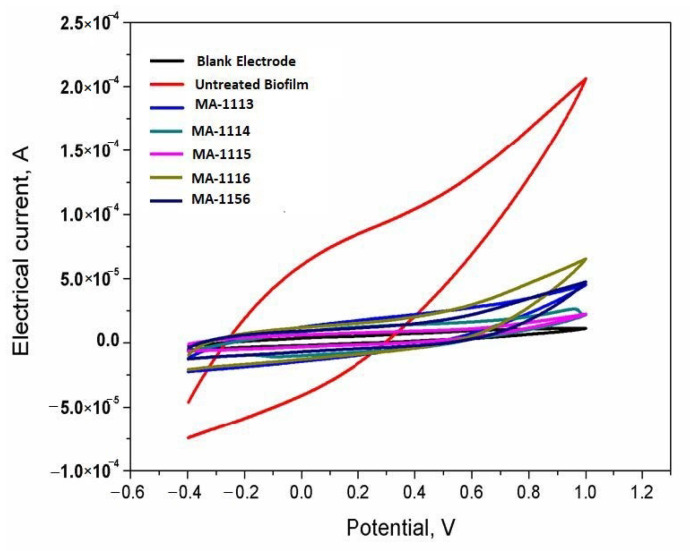
Bioelectrochemical responses of untreated vs. treated *S. aureus* biofilms with one concentration of each fluoroarylbichalcophenes.

The biofilm formed at the electrode surface (sensors surface) was used monitoring the *S. aureus* cell viability. From the obtained bioelectrochemcial signals, height of electric-current represents the high cell viability and fast electron transfer efficiency. Lowering the electric-current is reflecting the defects in biological functions of the living adhered cells at the electrode surface. Herein, the bioelectrochemical performances of the untreated *S. aureus* culture and fluoroarylbichalcophene treated cultures were recorded and analyzed. The results shown in Figure 7 demonstrate the inhibition effects on the formation of electrochemically active biofilm caused by the existence of fluorobichalcophenes in the microbial culture during the stage of biofilm formation.

As shown in Figure 7 the microbial-electrode interaction of *S. aureus* with the MnO_2_ nano-rods was measured in the presence of one sub-MIC concentration of the fluorobichalcophenes. To that end, the faradic current of treated *S. aureus* has much lower current values than the control untreated cells; 5 × 10^−5^ A for **MA-1156**, 2.5 × 10^−5^ A for **MA-1115**, 6.5 × 10^−5^ A for **MA-1116**, 5 × 10^−5^ A for **MA-1113** and 2.5 × 10^−5^ A for **MA-1114**. However, the untreated cells of *S. aureus* produced reasonable and significant higher faradic current reached about 2.5 × 10^−4^ A. This clearly points to the strong sensitivity of *S. aureus* to the tested fluoroarylbichalcophenes.

## 3. Discussion

The rapid and extensive development of antibiotic resistance in bacteria is a serious world-wide health problem. Therefore, continuous effort to develop novel and effective antimicrobial agents or increase the efficacy of the antibiotics currently in use by reducing the development of resistance in bacteria are highly needed [4]. Synthesized bichalcophene derivatives were found to have an efficient antimicrobial activity [8,9,10,11,12,22]. Bichalcophenes-based on thiophene and furan rings are recognized for their promising biological activity. The reported antimicrobial activity of the bichalcophenes can be attributed to the presence of sulfur and amidine functions [10,23]. In a previous study, bichalcophene derivatives were effective against both Gram-positive and Gram-negative bacteria like various broad-spectrum antibiotics; their mechanism(s) may possibly be through the inhibition of protein and/or nucleic acids synthesis [24], or bacterial DNA degradation [10].

In this study, all tested fluorobichalcophenes showed better antimicrobial activity than their corresponding parent compounds (1a and 1b) as the inhibition zone diameters of 1c to 1g compounds were from 15 to 20 mm against *S. aureus*. On the other hand, the inhibition zone diameters for parent compounds 1a and 1b were 14 and 15.5 mm, respectively. The compound **MA-1156** showed the highest activity inhibiting *S. aureus* growth at minimum inhibitory concentration value of 16 µM. A possible explanation for this result is that the antibacterial activity of these compounds may stem from the basic skeleton of the molecules as well as from the nature of the fluorine, sulfur, and/or oxygen heteroatoms substituents. This finding was inconsistent with a previous study on some bichalcophene and their aza-analogs [10].

The biological activity of bichalcophenes could be enhanced through introducing fluorine atoms to the phenyl ring [12]. The addition of extra thiophene ring in the former compound is the only difference between compound **MA-1156** and the other tested fluoroarylbichalcophenes. It cannot be denied that this additional thiophene rings is responsible for the impressive antibacterial activity. It was previously reported that compounds with thiophene rings were more active than those containing pyrrole or furan rings [8]. The low activity of **MA-1156** in the preliminary experiment using disc diffusion technique compared to the other tested compounds could be explained as; **MA-1156** may have lower diffusion ability or higher affinity to agar than the other tested fluoroarylbichalcophenes.

It was also reported that substitution of fluorine on the phenyl ring resulted in a significant improvement in the antiproliferative activity [25]. Similar findings were reported herein as the inhibition zone diameter and the MIC value are better enhanced when the fluorine atom is next to the bichalcophene moiety as in compounds **MA-1115** and **MA-1113** than their positional isomers **MA-1116** and **MA-1114**, respectively, in which the fluorine atom is next to the amidine group leading to improve the ability of compounds to inhibit the bacterial activity. This go along with a previous study on compounds **MA-1113** and **MA-1114** reported by Abousalem et al. [26] that could be explained by terms of the replacement of fluorine resulting in the apparently orthogonal effects of increasing molecular hydrophobicity and local polarity, thus altering their pharmacokinetic properties [25,27].

During the present research, different fluoroarylbichalcophene resistance was formed with *S. aureus* at one-fold MIC concentration against the compound **MA-1115** after three days’ incubation and **MA-1114** after two days’ incubation whereas the bacteria could not develop any resistance at higher concentrations. Similar findings for some monocationic pyridyl bichalcophene compounds have been reported previously against *E. coli* which could develop resistance to these bichalcophenes and tetracycline at one-fold MIC concentration after only one day of incubation, whereas at three-fold MIC concentration, the bacteria could not develop any resistance even after the 7th day of incubation against nearly all bichalcophenes [11]. General protection is provided by abundant multidrug resistant pumps (MDRs) that involve membrane translocations to extrude toxins from the cell [28,29,30]. The preferred substrates of most MDRs are synthetic hydrophobic cations such as quaternary ammonium antiseptics [31,32]. Mutational events that lead to target alteration or activation of efflux pump mechanisms are the most reasonable explanation for their resistance [33].

From scanning electron microscopy, the untreated *S. aureus* micrograph showed normal morphology and typical grapelike cluster arrangement of *S. aureus* cells. While cells exposed to each of fluoroarylbichalcophenes undergo some ultra-structural changes as decreasing the number and size of cells around 0.5 µm, appearance of some deformities in the external shape of cell up to lyses of some cells. Moreover, the appearance of the bacterial cells treated with **MA-1116** and **MA-1113** compounds as streptococci or linear arrangement could be attributed to the division of cells in one direction. That goes along with a previous study of *Staphylococci* treated with cloxacillin where the progressive separation of the bacteria was in a manner fundamentally similar to that seen in streptococci [34].

The SDS-PAGE analysis showed qualitative changes in protein profile of *S. aureus* after treatment with the tested fluoroarylbichalcophenes. Disappearance of protein bands have been attributed to the degradation of proteins by the antimicrobial agents whereas the appearance of new bands and/or the increasing in bands intensity could be due to the synthesis of responsive proteins that might resist the stress or help the bacteria to survive in stressful conditions [35]. Low molecular weight protein bands may be evolved from degradation of high molecular weight proteins [36]. The antimicrobial activity of these bichalcophenes could occur because the synthesis of protein and/or nucleic acids is inhibited [24,37,38].

The minimum percentage of viability results for *S. aureus* cells treated with **MA-1156** were recognized at all tested concentrations where cells were unable to cleave WST-1 within 1 h of complement-mediated lysis so no formazan was formed. In contrast, the cells treated with **MA-1114** showed the maximum percentage of viability as *S. aureus* cells were able to cleave WST-1 and form the formazan product at all except 128 µM (1.7%). WST-1 is cleaved by all living, metabolically active cells only, but not by dead cells. The amount of formazan generated is directly proportional to the cell number over a wide range, using a homogeneous cell population. Activated cells produce more formazan than resting cells, which could allow the measurement of activation. These properties are all consistent with the cleavage of WST-1 only by active cells [39]. Due to the microbial-catalytic oxidation of degradable organic compounds, electrons transmitted to a molecule other than oxygen under anaerobic conditions via the electron transport chain (ETC). Accepting the electrons results in the production of electrical current directly proportional to the total number of viable microbial cells and their activity. Dormant or dead cells, in contrast, have no electrochemical activity [40]. In the end, online monitoring of microbial responses and cell viability could be enabled to differentiate the living from the dead microbes [41,42]. Herein this study, the treated *S. aureus* cultures with the synthesized fluoroarylbichalcophenes, produce lower bioelectrochemical signals ranging from 2.5 to 6.5 × 10^−5^ A than the untreated cell which recorded 2.5 × 10^−4^ A. This sharp decrease points to the ability of the tested fluoroarylbichalcophenes to inhibit the direct electron transfer from viable bacterial cells to the carbon electrode. A previously measured *S. aureus* activity was electrochemically using a double-mediated bioelectrochemical system. The electrochemical signals were obtained only from the metabolically active cells, whereas an oxidation peak current was observed at about 0.6 V vs. Ag/AgCl, while the metabolically inactive cells showed almost no bioelectrochemical responses [43]. In the light of previously mentioned results, it could be concluded that the antibacterial activity of bichalcophene derivatives against *S. aureus* is enhanced due to the incorporation of fluorine atom.

## 4. Materials and Methods

### 4.1. Tested Bichalcophene Derivatives and Bacteria

Two non-fluorinated parent compounds; **1a** and **1b** and five fluorophenylbichalcophenes; **1c**–**1g** (Figure 1) were available from previous studies [10,12] and provided by Professor M. A. Ismail. Their chemical names: **1a** (4-(2,2′-bithiophene-5-yl) benzamidine); **1b** (4-(2,2′-bifuran-5-yl) benzamidine); **1c** (4-(2,2′-bithiophen-5-yl)-2-fluorobenamidine); **1d** (2-fluoro-4-(5-(thiophen-2-yl) furan-2-yl) benzamidine); **1e** (3-fluoro-4-(5-(thiophen -2-yl) furan-2-yl) benzamidine); 1f (4-(2,2′-bifuran-5-yl)-3-fluorobenzamidine); 1g (4-(2,2′-bifuran-5-yl)-2-fluorobenzamidine). The tested compounds were liquefied at 10 mM concentration in 1 mL absolute DMSO. *S. aureus* used in this study was multi-drug resistant bacteria as it was resistant to Oxacillin, Ciprofloxacin, Methicillin, Levofloxacin, Ofloxacin, Erythromycin and Ampicillin and sensitive to Cefoxitin and Gentamicin.

### 4.2. Detecting the Antimicrobial Activity of the Tested 2,2′-Bichalcophene Derivatives

Disc agar diffusion technique described by Bauer et al. [44] was used to evaluate the antimicrobial activity of the bichalcophene compounds **1a**–**1g** against *S. aureus*. The tested compounds were liquefied at 10 mM concentration in DMSO. Mueller-Hinton agar plates were inoculated separately with 10^7^ CFU/mL of bacterial cultures and regularly spread on the whole surface of each plate. The 5 mm diameter sterile discs were saturated with 10 µL of the tested bichalcophene compounds and placed on LB plates inoculated with the tested microorganisms. The plates were incubated for 24 h at 37 °C, after that inhibition zones were measured in millimeters and compared with a negative control 10% DMSO. Each assay in this test was conducted in three replicates.

### 4.3. Determination of MIC of the Tested 2,2′-Bichalcophene Derivatives

The MIC of bichalcophene derivatives was measured according to the processes described by Clinical and Laboratory Standards [45]. Different concentrations (0–128 μM) of the tested bichalcophenes dissolved in DMSO were added independently to LB broth medium which was previously autoclaved. For the current assay, prepared LB culture of *S. aureus* was chosen. A 20 μL seed culture of the tested organism having nearly 5 × 10^4^ colony forming units (≈0.5 OD) was used as an inoculum for testing the bichalcophene derivatives. Respective blanks (culture and broth alone) were preserved, subsequently. All tubes were incubated overnight at 37 °C and measured at 600 nm optical density. Experiments were performed as triplicates.

### 4.4. Detection of Fluoroaryl-2,2′-Bichalcophenes-Resistant Variants

In order to evaluate the development of resistance in *S. aureus* against fluoroarylbichalcophenes, growth assays were performed in the presence or absence of each bichalcophene derivative. 20 mL of cells were centrifuged, and the cells were re-suspended in 2 mL of MHB (Mueller-Hinton broth). The inoculum density was adjusted to 10^8^ CFU/mL. Different concentrations of each tested bichalcophene derivative (3×, 2×, 1× MIC) were added. All tubes were incubated at 37 °C and the absorbance was recorded at 600 nm daily over a period of seven days [33].

### 4.5. SDS-PAGE of *S. aureus*

*S. aureus* samples treated with the tested fluoroarylbichalcophene derivatives and the untreated sample (control) were cultured in LB broth media at 37 °C and 120 rpm for 24 h. The bacterial cells were harvested by centrifugation at 10,000 rpm for 5 min. The pellets were homogenized in phosphate buffer (0.6 M, pH 6.8) using glass beads and FastPrep^®^-24 homogenizer and then centrifuged at 10,000 rpm for 5 min for protein isolation [46]. Ten µg protein samples were boiled into 2× sample buffer (10 mL Distilled Water, 2.5 mL Tris HCl pH 6.8, 2 mL glycerol, 4 mL of 10% SDS and 1 mL β-mercaptoethanol) for 2 min. Around 20 µL treated proteins were loaded over acrylamide gel. Acrylamide gel was prepared according to Laemmli [47] from two layers; 4% stacking gel on top of 12% separating gel. After electrophoresis at 100 V for 2 h, a gel was overnight stained in Commassie brilliant blue R_250_ and visualized by soaking in destaining solution on shaker for some hours. The gel was documented and analyzed using the gel analyzer3 program.

### 4.6. SEM

Cells of *S. aureus* treated with the sub-MIC of each tested fluoroarylbichalcophene, **MA-1156** (4 µM), **MA-1115** (8 µM), **MA-1116** (16 µM), **MA-1113**, and **MA-1114** (32 µM) were subjected to SEM microscopy (JSM-6510 L.V., Tokyo, Japan). The bacteria were incubated for 12 h in incubator at 37 °C in LB broth containing the sub-MIC of fluoroarylbichalcophenes besides the untreated one (control). The pellets of bacterial cells were harvested from 10 mL of each sample by centrifugation at 5000 rpm for 10 min and processed according to Hartmann et al. [48]. The specimens were coated with gold-palladium membranes and examined by SEM microscope using 30 KV at EM Unit, Mansoura University, Egypt.

### 4.7. Cell Viability Determination Using WST-1 Reagent

The cell density of the selected strain *S. aureus* was quantified by measuring the viability of the cells using WST-1 reagent according to Mosmann [49,50]. Starting at 0-incubation time, whereas 4 mL sterilized LB media amended different concentrations (0, 4, 8, 16, 32, 64, 128 µM) for all fluoroarylbichalcophenes. A 1 mL inoculum seed culture of *S. aureus* was added and incubated in a shaker at 650 rpm for 30 min at 37 °C. Consequently, 20 μL of 10 mM WST-1 solution was added per tube then shacked for (1–2) min, afterwards, the absorbance at 450 nm was measured and considered as zero time. Further incubation of the treated microbial cultured with fluorobichalcophene derivatives and WST reagent for another 30 min before re-measuring the absorbance at the same wavelength [49]. The overall cell viability percentage was calculated as the following equation:

Cell viability% = (absorbance value of treated cells)/(absorbance value of untreated cells) × 100

### 4.8. Bioelectrochemical Measurements

All electrochemical experiments were performed utilizing a three-electrode Gamry Potentiostat/Galvanostat/ZRA G750 system known as a voltammetry. The working electrode was electrochemically activated by applying five cyclic scans from −0.4 to 1.0 V vs. Ag/AgCl, with the scan rate of 50 mV/s in phosphate buffer (pH 7.4) as a supporting electrolyte. The basic concept of this assay relies on the direct electron transfer from living microbial cells that physically adhere to the electrode surface [39,51].

To investigate the response of *S. aureus* cells to the targeted fluorobichalcophenes, a suspension of *S. aureus* was incubated with one sub-MIC concentration of each compound; 4 µM of **MA-1156**, 8 µM of **MA-1115**, 16 µM of **MA-1116** and 32 µM of both **MA-1113** and **MA-1114** at 37 °C for two weeks to form biofilm by attaching microbial cells at the screen printed electrode and measure current by measuring the bioelectrochemical responses of the treated cells. The cyclic voltammograms were recorded at different time intervals to monitor the online growing of the biofilms on the modified electrode surfaces and to detect the effect of the fluoroarylbichalcophenes on the electron transfer from the adhered bacterial cells and the working electrode surface. The resulting curve is called a voltammogram, whereas electric potential and electric currents are plotted on the X and Y axes, respectively.

### 4.9. Statistical Analysis

For the goals of this study, all assays were performed in triplicate in three independent experiments. Statistical analysis results were expressed as means ± S.E. (standard error).

## 5. Conclusions

**MA-1156** was the most effective monocationic fluoroaryl bichalcophene compound for inhibiting the microbial growth and could be used as an effective antimicrobial agent for living tissues.

## Figures and Tables

**Table 1:** Inhibitory effect of monocationic bichalcophenes on *S. aureus*.

Tested Compound (10 mM)	Inhibition Zone Diameter (mm)
**MA-0944 (1a)**	14 ± 0.2
**MA-0947 (1b)**	15.5 ± 0.0
**MA-1156 (1c)**	15 ± 0.2
**MA-1115 (1d)**	20 ± 0.2
**MA-1116 (1e)**	16 ± 0.2
**MA-1113 (1f)**	16 ± 0.1
**MA-1114 (1g)**	20 ± 0.2

**Table 2:** Determination of the MIC, and MBC (µM) of the tested fluoroarylbichalcophene derivatives (**1a**–**1g**) against *S. aureus*.

Compound	MIC (µM)	MBC
**MA-0944 (1a)**	128	not detected
**MA-0947 (1b)**	128	not detected
**MA-1156 (1c)**	16	16
**MA-1115 (1d)**	32	32
**MA-1116 (1e)**	64	128
**MA-1113 (1f)**	128	128
**MA-1114 (1g)**	128	128

## Data Availability

The data presented in this study are available on request from the corresponding author.
